# The Nature of the Low-Temperature Crossover of Water in Hard Confinement

**DOI:** 10.1021/acs.jpcb.3c00747

**Published:** 2023-05-25

**Authors:** Yael Beilinson, Verena Schiller, Julia Regentin, Jorge H. Melillo, Anna Greenbaum, Tatiana Antropova, Silvina Cerveny, Michael Vogel, Yuri Feldman

**Affiliations:** † Department of Applied Physics, The Hebrew University of Jerusalem, Edmond J. Safra Campus, Jerusalem 9190401, Israel; ‡ Institut für Physik kondensierter Materie, Technische Universität Darmstadt, Hochschulstraße 6, 64289 Darmstadt, Germany; § Donostia International Physics Center (DIPC), Paseo Manuel de Lardizabal 4, 20018 San Sebastian, Spain; ∥ The Hebrew University of Jerusalem, Racah Institute of Physics, Edmond J. Safra Campus, Jerusalem 9190401, Israel; ⊥ Grebenshchikov Institute of Silicate Chemistry, Russian Academy of Sciences, Makarova emb., 2, Saint-Petersburg 199034, Russia; # Centro de Física de Materiales (CFM CSIC/EHU) - Material Physics Centre (MPC), Paseo Manuel de Lardizabal 5, 20018 San Sebastian, Spain

## Abstract

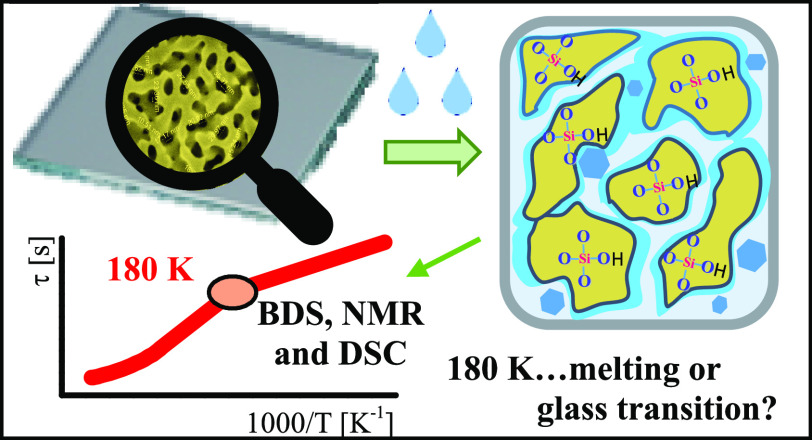

The dynamics of water confined in mesoporous MIP (2–3 nm pores in size) with silica gel (secondary silica; further, the abbreviation SG will be used) and MAP (10–35 nm pores in size) without SG borosilicate glasses have been studied by broadband dielectric spectroscopy (BDS), nuclear magnetic resonance (NMR), and differential scanning calorimetry (DSC). MIP samples contain secondary silica inside the pores and provide a confinement size of about 2–3 nm, whereas MAP samples are free of secondary silica and provide a confinement size of about 10–35 nm. It is shown by BDS and NMR techniques that water exhibits a dynamic crossover of around 180 K when it is confined in MIP samples. By contrast, water confined in larger pores (MAP) does not exhibit any changes in its relaxation behavior. It is also shown that the crossover temperature depends on the hydration level (the higher the hydration level, the lower the crossover temperature). Below the crossover temperature, we find that water reorientation is isotropic (NMR) and that the temperature-dependent dielectric relaxation strength (BDS) follows the tendency expected for a solid-like material. In contrast, water reorientation is related to long-range diffusion above the crossover temperature, and the dielectric relaxation strength follows the tendency expected for a liquid-like material. Furthermore, the calorimetric results are compatible with crossing a glass transition near 180 K. Finally, the results are discussed within the Gibbs–Thomson model. In this framework, the crossover could be related to ice crystals melting.

## 1 Introduction

Water is considered a crucial element in organic and inorganic complex systems. Life, as we know it, would not exist without water – water is essential for all known forms of life; therefore, understanding its dynamic properties is very valuable.^1−3^ However, understanding how the molecular properties of water translate into its ability to solvate a molecule or interact at a molecular interface – the hydration layer – with hydration centers is still debated.^4,5^ Critical differences exist between the structure and dynamics of water in bulk and at the interface. For this reason, water in the hydration layer can be considered confined water. Yet water can be confined in several ways, from inorganic zeolites to microemulsions. Therefore, will there be universality in water behavior or not? Is there a difference between whether that confinement is “hard” (water in the pores of a solid material) or “soft” (hydration shell of a solute or water at a biological interface)? The effects of confinements on the water structure and dynamics, hard or soft confinements, directly influence the thermodynamic state of water. Current research addresses whether there is universality in the behavior of water for all confinement types and, if so, what is the mechanism of such behavior.

**Figure 1:**
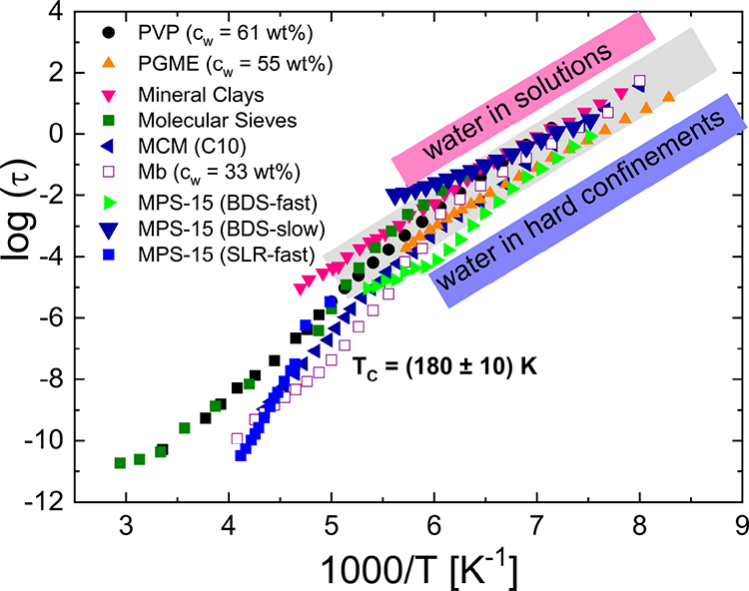
Temperature dependence of relaxation time of water in aqueous solutions of poly(vinylmethylether) (PVP) and propylene glycol monomethyl ether (PGME), hard confinement systems (mineral clays, MCM-41 and mesoporous silica (MPS, 2.6 nm)^6^) and hydrated myoglobin^5^ (Mb). *c*_w_ represents the water content of each sample, and *T*_c_ is the temperature where the cross-link is produced. For water in MPS, faster and slower water processes were observed where BDS represents broadband dielectric spectroscopy and SLR is spin–lattice relaxation measurements.

The specific dynamic pattern of confined water studied here is usually observed in the dynamic crossover of dielectric relaxation times from liquid-like behavior (i.e., Vogel–Fulcher–Tammann (VFT) dependence) toward localized or solid-like (i.e., Arrhenius dependence) motions with decreasing temperature (see Figure 1).

**Figure 2:**
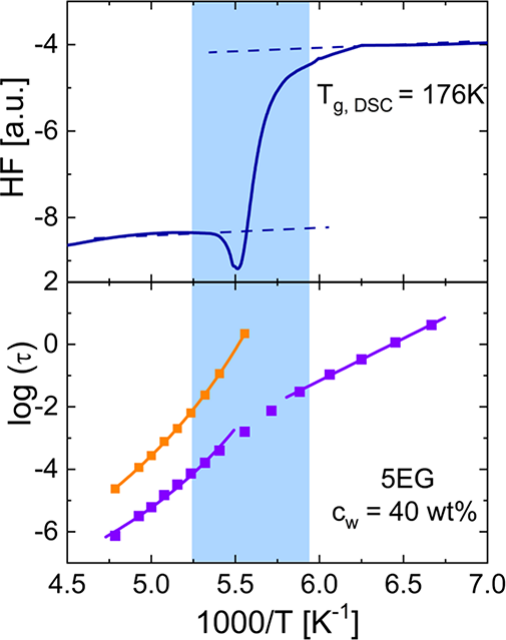
Heat flow measured by differential scanning calorimetry (DSC) during heating of the aqueous solution of ethylene glycol pentamers (5EG, water content, *c*_w_ = 40 wt %) at a rate of 10 K min^–1^ (upper graph). Temperature dependence of the relaxation times for the same sample. The orange symbols indicate the relaxation times for the α-relaxation, whereas the purple symbols represent the relaxation times for the water molecules^5^ (bottom graph).

As one can see from Figure 1, the dynamic crossover takes place in various hard or soft confinements (solutions of biological and non-biological solutes). The crossover observed in water solutions is usually attributed to the thermodynamic properties of the investigated liquid.^7,8^ The crossover temperature is detected near the glass transition temperature (*T*_g_) of the corresponding solution determined by calorimetric measurements (see Figure 2). However, the glass transition detected corresponds to the solute affected by water. Below *T*_g_, water molecules are trapped in the frozen matrix, and water motions are restricted and similar to those corresponding to a secondary β-relaxation in other glasses. Therefore, the temperature dependence of their relaxation times is Arrhenius-like. By contrast, the crossover in the temperature dependence of the relaxation times is produced at *T*_c_ = (180 ± 10) K independent of the matrix for water under rigid confinements.

By contrast to water solutions, in hard confinement systems, a *T*_g_ related to water has not been detected by standard calorimetric measurements where only crystallization and melting of water are seen.^9−12^ Considering other calorimetric techniques, such as adiabatic measurements, Oguni et al.^13^ claimed that two glass transitions can be distinguished for confined water in MCM-41. However, this view was discussed later by Johari et al.,^14,15^ indicating that the feature detected is not a glass transition but rather a decay of ice-like structures during the heating. There is also a report by Kittaka et al.^16^ for water confined in MCM-41 (pore size 2,1 nm) where a broad endothermic peak is observed at approximately 190 K, and it was assigned to the melting of frozen and/or glassy water.^16^ Thus, despite the Oguni et al. observation,^13^ there are no other reports on the glass transition of confined water.

At temperatures lower than *T*_c_, the *T* dependence of the water relaxation time follows an Arrhenius behavior, which becomes universal for water under hard and soft confinements when the water content is sufficiently high. At such high water concentrations, most water molecules are expected to be surrounded by other water molecules, and the solute’s or interface presence should influence less their relaxation. In this situation, for both types of confinements, the crossover is produced in the same temperature range – around 180 K in the so-called “no man’s land”^17^ temperature range of bulk water. Although there are a few attempts to explain the origin of this crossover, the final mechanism is still under debate.^4,18−23^ However, in some mesoporous silica, two water fractions with distinguishable dynamics were observed and attributed to water in the interfacial layer and pore interior, respectively.^6^

Recently many attempts were focused on investigating the water in hard and soft confinements to explain the origin of the crossover phenomenon studied in porous silica matrixes,^18,24−27^ clays,^28^ or hydrated proteins.^29,30^ The freezing and melting temperatures of confined water are shifted, Δ*T_f_* = *T_f_*(bulk) – *T_f_*(pore), depending on the pore radius, as described by the modified Gibbs–Thomson model, GT, in eq 1,^31^
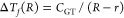
1Here, *C*_GT_ is considered as a constant and depends on the actual system under investigation, i.e., the properties of the fluid, the fluid/solid interface, the density of the solid, the bulk enthalpy of fusion, and the temperature; *R* is the radius of the pore; *r* represents the thickness of a liquid-like layer at the relevant melting temperature.^32^ The scenario proposed here is that the crossover temperature is related to the confined water phase transition and corresponds only to the size of the confinement.^33^ However, this model cannot explain the uniqueness of the experimentally observed crossover temperature for a very broad class of materials of different natures of confinement.

Therefore, the main goal of this research is to study the dynamics and structure of confined water as a function of the topology and hydration level of the matrix. For this purpose, water in various confined types should be studied comprehensively by combining several experimental techniques that reveal molecular dynamics. Techniques appropriate to the task are broadband dielectric spectroscopy (BDS), nuclear magnetic resonance (NMR), and differential scanning calorimetry (DSC). The first two techniques reveal the dynamics of confined water molecules, and DSC provides information on the thermodynamic state of water at a specific temperature. In this article, the experiments will focus on water in hard and soft confinements as the first part of comprehensive research.

## 2 Methods

### 2.1 Synthesis of the Porous Glasses

Samples of porous glasses of two types (MIP and MAP) with different pore space structures were studied in the form of flat-polished plates with a size of 10 × 10 × (1–1.5) mm^3^. The abbreviations MIP and MAP mean, respectively, “microporous” glass and “macroporous” glass according to the terminology of Zhdanov.^34^ By the IUPAC classification,^35,36^ the studied glasses belong to mesoporous materials.

Sodium borosilicate glass with composition (according to synthesis, mol %): 8Na_2_O, 22B_2_O_3_, 70SiO_2_, subjected to heat treatment at 550 °C for 144 h to form a two-phase structure, was used to manufacture porous glasses. Porous glasses were obtained from the chemical etching of two-phase glass by the standard procedure.^37^

To obtain a porous glass of the MIP type, two-phase glass samples were leached in 3 M HCl solution by boiling followed by washing in distilled water at room temperature and drying at 120 °C for 1 h in an air thermostat. The secondary silica (SG), which is present in the pores of the MIP type porous glass, can be considered as a soft confinement for water molecules mainly adsorbed at the geminal and vicinal silanol groups (SiOH) of non-chemically bonded SG^38−40^ rather than siloxane bridging oxygen groups (Si–O–Si), which are more typical for the glass matrix surface.^41,42^

To obtain a porous glass of the MAP type, MIP-type samples were processed in 0.5 M KOH solution at 20 °C followed by washing in distilled water at room temperature and drying at 120 °C in an air thermostat. As a result, secondary silica was completely removed from the pore space of the MIP glasses.

### 2.2 Broadband Dielectric Spectroscopy (BDS)

Dielectric measurements in an extended frequency range of 10^–2^–10^9^ Hz were performed by the broadband dielectric spectrometer BDS 80, an Alpha Impedance Analyzer, and an RF spectrometer, based on an Agilent 4291 RF impedance analyzer, Novocontrol, with automatic temperature control by the QUATRO Cryosystem using liquid nitrogen with a precision of 0.01 °C.^43^ The accuracy of the complex dielectric permittivity was estimated to be better than 3%.^44,45^ The samples were measured at intervals of 3 °C upon heating them from 140 K to 350 K. In addition to this routine, porous glass samples were held at 530 K after the heating procedure for 3 h to evaporate physically adsorbed water from the sample to get dry glass weight. The diameter of the electrodes for the measurements was 12 mm, and the thickness of the plate samples was 1.457 mm.

### 2.3 Calorimetric Characterization

Differential scanning calorimetry (DSC) measurements were performed using Q2000 TA equipment in the temperature-modulated mode (TMDSC). The total heat capacity (*c*_P_) is equivalent to standard DSC, the reversing signal (R*c*_P_) provides information on heat capacity and melting, and the non-reversing signal (nR*c*_P_) shows the kinetic events, such as the crystallization. In TMDSC, a periodic temperature perturbation is superimposed on linear heating or cooling. TMDSC experiments were carried out with an amplitude *T*_A_ = 0.16 K using a heating rate of 1 K/min. The modulation period was 60 s (ω = 0.105 rad s^–1^). Before hydration, samples were cut using a pen diamond to a weight of about 10 to 15 mg. Samples were prepared in hermetic pans.

### 2.4 Nuclear Magnetic Resonance (NMR)

The ^2^H NMR experiments were performed on a home-built spectrometer at a ^2^H Larmor frequency of ω_L_ = 2π·46.1 MHz using a 90° pulse length of ca. 2 μs. For ^2^H spin–lattice relaxation (SLR) measurements, we use the saturation-recovery sequence together with solid-echo detection. In ^2^H stimulated-echo (STE) experiments, three pulses divide the experimental time into two fixed evolution times *t_e_* and a variable mixing time *t_m_*. Here, a fourth pulse is applied to refocus the stimulated echo outside the dead time of the receiver and an appropriate phase cycle is applied to eliminate unwanted single and double quantum coherences.^46^^1^H field-cycling relaxometry (FCR) studies were conducted to measure the frequency dependence of the ^1^H spin–lattice relaxation time. The used setup and the data analysis were described in some detail in previous works.^47,48^ Finally, we used ^1^H static field gradient (SFG) NMR to measure the self-diffusion coefficients *D* of water. These measurements were performed at a Larmor frequency of ω_L_ of 91 MHz and a strength of the magnetic field gradient g of 139 T/m. The 90° pulse length amounted to ca. 1 μs. Further information about the SFG setup and experiments can be found in previous publications.^49,50^

### 2.5 Materials and Characterization

**Figure 3:**
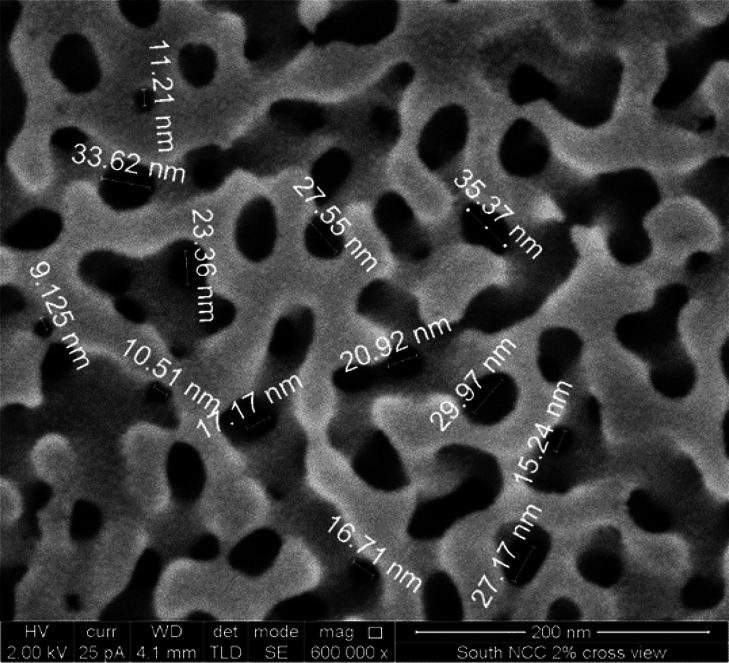
SEM image of the interconnected glass porous structure.

Samples of porous glasses MIP (microporous) and MAP (macroporous) were selected for the experiments. This combination of structures provides nanosized geometrical confinement and allows studying water hydrated by rigid/hard (glass) and soft (silica gel, SG) matrices. Further, the glass could be cleaned from the silica gel matrix by alkali treatment (MAP). Therefore, the response from water bound with various hydration centers of glass and silica gel could be distinguished. The size of the glass pore of MAP-type varies between 10 and 35 nm, while the confinement size in MIP-type samples is 2.5–3 nm. The SEM image of the interconnected pore structure is shown in Figure 3. Silica gel fine structure cannot be resolved by this technique.

The pore space structure parameters of the MIP and MAP glasses were studied by the method of low-temperature nitrogen desorption. The equilibrium adsorption and desorption isotherms of nitrogen at 77 K and the pore size distribution are given in the work.^45^ The average values of porosity, specific surface area and diameter of the pores are shown in Table 1.

**Table 1:** Structure Parameters of the Porous Glasses under Study^37^

porous glass	porosity *W*, cm^3^/cm^3^ (%)	specific surface area *S*, m^2^/g	mean pore diameter *D*, nm
MIP	0.29 (29)	164	3
MAP	0.59 (59)	73	25

**Figure 4:**
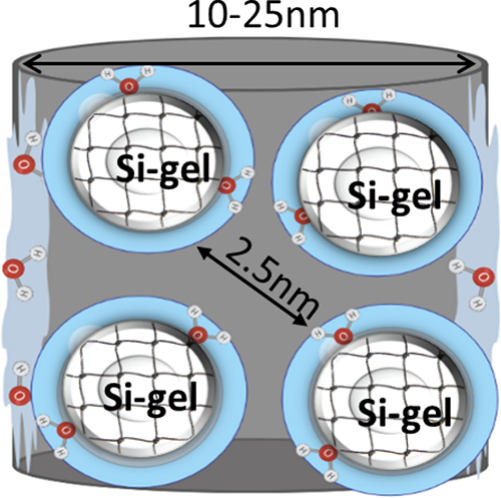
Schematic picture of the studied MIP-type samples. The glass channels diameter varies between 10 and 25 nm. Confinement size before hydration is *d* ≈ 2.5–3 nm.

To estimate the pore sizes in secondary silica (SG) filling the pore space of MIP-type glasses, we consider experimental data^45^ obtained by studying the parameters of the porous structure of various porous glasses, including MIP-type and MAP-type samples similar to those used in our work. Additional treatment of MIP glass in an alkaline solution leads to an increase in the average diameter of mesopores from 2.5–5 to 20–30 nm due to the dissolution and removal of SG from the pore space. At the same time, the volume of micropores with an average diameter of approximately 0.6 nm, located inside the SG globules, becomes insignificant. The schematic picture of MIP samples with secondary silica globules inside the porous glass is presented in Figure 4.

The studied porous glasses are characterized by the same surface groups (meaning the surface of the walls of the silica skeleton and the surface of the globules of secondary silica (SG) inside the pore space), which are marked on any hydrated silica surface.^39,51^

Silica gel itself is also a porous material, and there is the probability that some ice crystals could grow inside silica gel particles (Figure 4). During hydration, as discussed, silica gel swells and reduces the size of the confinement. The evaluated confinement for all samples is about 2–3 nm – this is close to the size limit of water crystallization.^52,53^

### 2.6 Sample Hydration and Preparation

The samples were weighted prior and immediately after the dielectric measurements. The water fraction *h* = *M*_w_/*M*_gl_ was calculated based on the masses *M*_w_ and *M*_gl_ of the physically absorbed water and the dry glass, respectively. The calculated water fractions are presented in Table 2. Note that the alkali-treated sample without silica gel (SG) (MAP) has a much lower water content than the samples containing SG (MIP), which indicates that SG is the main water adsorbent.

**Table 2:** Hydration of Porous Glass Samples^a^

		vapor under saturated salt solutions		
hydration, %	Vapor H_2_O (MIP)	NaCl (MIP)	Mg(NO_3_)_2_ (MIP)	Mg(NO_3_)_2_ (MAP)
sample’s abbreviation	MIP_23	MIP_13	MIP_11	MAP_2
by weight (h)	23.8	13	11	2

a Holding samples at atmosphere with various humidities at 23 °C for 2 days.

Following the same procedure described above, DSC samples were hydrated for 15 days under vapor-saturated salt solutions to obtain water fractions of 4.8 and 11.1 wt %. We will refer to these samples as MIP_4.8 and MIP_11, respectively.

Furthermore, analogous procedures were employed for the hydration of the NMR samples. A ^1^H NMR sample was prepared using H_2_O and the porous glass with SG. It has a water content of *h* = 10% and will be denoted as MIP_10. Four ^2^H NMR samples were prepared utilizing D_2_O and porous glasses with or without SG. We obtained water fractions of 10% and 16% for MIP and 3% and 16% for MAP. We will refer to these samples as MIP_10D, MIP_16D, MAP_3D, and MAP_16D, respectively. All hydrated materials were filled into NMR tubes, which were flame-sealed immediately afterward.

## 3 Results and Discussion

### 3.1 Dielectric Spectroscopy

**Figure 5:**
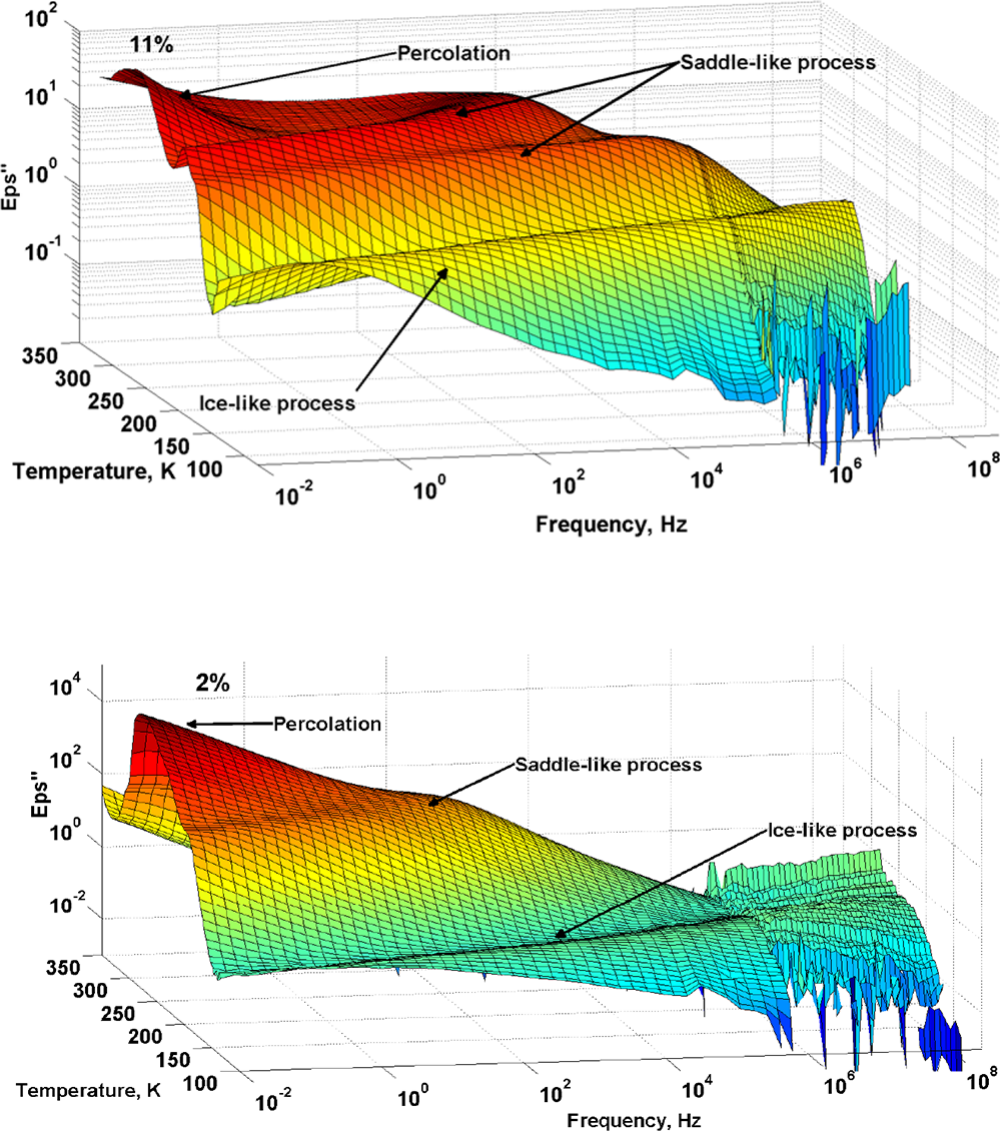
3D plot of the frequency and temperature dependence of the dielectric losses for the porous glass MIP_11 (upper panel) and MAP_2 (bottom panel), correspondingly.

Typical 3D plots of the frequency and temperature dependence of the dielectric loss for the porous glass matrix with (MIP_11, upper panel) and without (MAP_2, lower panel) SG are presented in Figure 5. The dielectric spectra show several distributed relaxation processes.

The typical relaxation process assigned to the ice-like process^25,54^ is observed in the low temperature region from 140–220 K. This process is also observed in many works and so-called as the first process or ν-process, or main.^4,5,55,56^ This relaxation process has been reported earlier for different hydrated heterogeneous systems^24,57^ reflecting confined water molecules.

The process in the temperature range of 180–350 K has a specific saddle-like shape. The saddle-like process is usually observed in different hydrated heterogeneous materials^58,59^ and assigned to the cooperative relaxation of water at the interface, where the balance between defect formation and water reorientation in its vicinity takes place.^59^ This relaxation process is thought to be a kinetic transition due to water molecule reorientation in the vicinity of defects.^54^ Note that the sample without SG has only one saddle-like process while the sample, which contains SG, has two, reflecting the coexistence of two confinement sizes.

The percolation relaxation process is well marked in the temperature range of 220–270 K. This process is due to the apparent dipole moment excitation within the developed fractal structure of the connected pores.^24,57,60^ This excitation is associated with the self-diffusion of the charge carriers in the porous net. Note that as distinct from dynamic percolation in ionic microemulsions, the percolation in porous glasses appears via the transport of the excitation through the geometrical static fractal structure of the porous medium.

**Figure 6:**
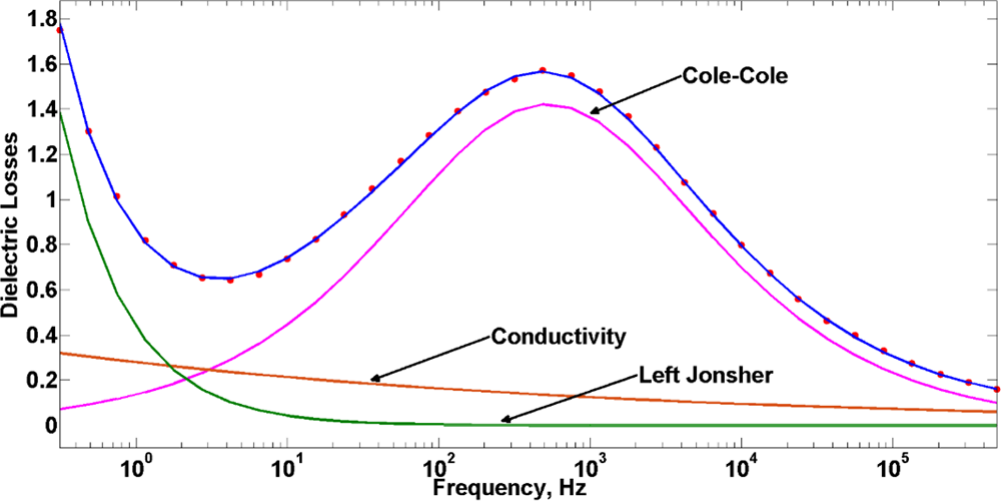
Dielectric losses in dependence on frequency (sample MIP_11 at 178 K). Measured data – red dots, fitting function – blue line, and individual fit contributions – other lines.

For a quantitative analysis of the dielectric spectra of the confined water at low temperatures, a superposition of phenomenological Cole–Cole(CC) functions,^61^ a low-frequency Jonscher term,^62^ and a conductivity term was used.^63^ The general fitting function is given by eq 2,
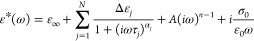
2where ε*(ω) is the measured complex dielectric permittivity, the parameter τ_*j*_ defines the relaxation time of the *j*th process, *i*^2^ = −1, ω *= 2*π*f*, where *f* is the frequency, Δε_*j*_ = ε*_s_* – ε_∞_ defines the dielectric strength of the *j*th relaxation process, with ε*_s_* and ε_∞_ denoting the extrapolated low-frequency and high-frequency permittivity limits, respectively; the parameter α*_j_* describes the symmetric broadening of the corresponding relaxation process, *A* is the amplitude of the Jonscher term, with exponents 0< *n* <1, σ_0_ is the *dc*-conductivity, and ε_0_ = 8.85·10^–12^ F/m is the dielectric permittivity of vacuum. The fitting was carried out using the homemade DATAMA program.^64^ A typical example of the measured data and the fitting functions is shown in Figure 6.

**Figure 7:**
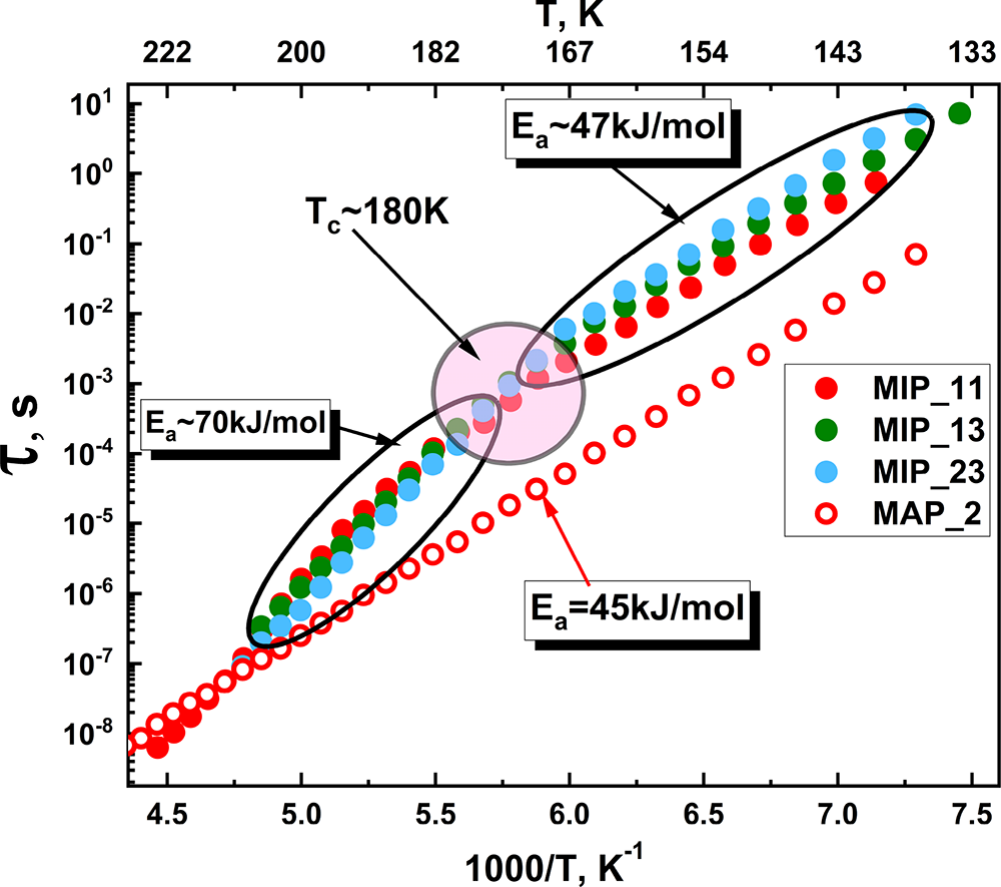
Relaxation times of ice-like process versus inversed temperature for the measured samples with various hydration levels. Filled symbols represent the low-temperature relaxation process of water in the silica gel structure (MIP) and empty symbols are that at the glass surface (MAP). The process related to the glass surface does not exhibit a crossover (empty red symbols).

Figure 7 presents the Arrhenius plot of the relaxation times for the ice-like processes (first, ν/main) of all studied samples.

As mentioned above, the MIP samples have two types of hydration centers for water adsorption – at the hard silica glass matrix surface and the SG structure. According to existing concepts (see, for example, refs (38, 41, 65−67) and reviews in them), the centers of water sorption on the silica surface are the isolated, geminal, and vicinal silanol groups (SiOH), which are located mainly in places with significant surface curvature (such are the narrow pores between SG globules). Along with SiOH groups, the silica surface also contains less reactive hydrophobic siloxane bridging oxygen groups (Si–O–Si), which are more typical for the glass matrix surface. During hydration of porous glass, one should expect the formation of a coordination bond of water molecules with the active centers of the silica surface of the glass matrix and the hydrogen bonds (HBs) of the following types (O_water_···H_water_), (O_water_···H_silanol_), and (O_siloxane_···H_water_), which are influenced by the surface density of SiOH groups, significantly depending on humidity, and the limited geometry of the nanoporous structure (reducing the size of the pores contributes to a stronger binding of hydrous species). SG is a strongly hydrophilic substance, and the water preferably adsorbs inside its porous structure. Only a tiny amount of water can be separately adsorbed at the inner surfaces of the glass matrix. The measurement of MAP_2 was also performed to confirm this. It shows a single relaxation with a temperature-independent slope in the Arrhenius plot (see Figure 7, empty red symbols). The formation of the ice-like (first, ν/main) water structure is strongly dependent on the amount of water covering the pore surface. The activation energy *E*_a_ of the relaxation process is dependent not only on the water content but also on the microstructure of the pore surface and the amount of SG inside the pores. The MAP sample contains water molecules in the inner pore surface, and they form the ice-like structures confined between the pore and air gap.

**Figure 8:**
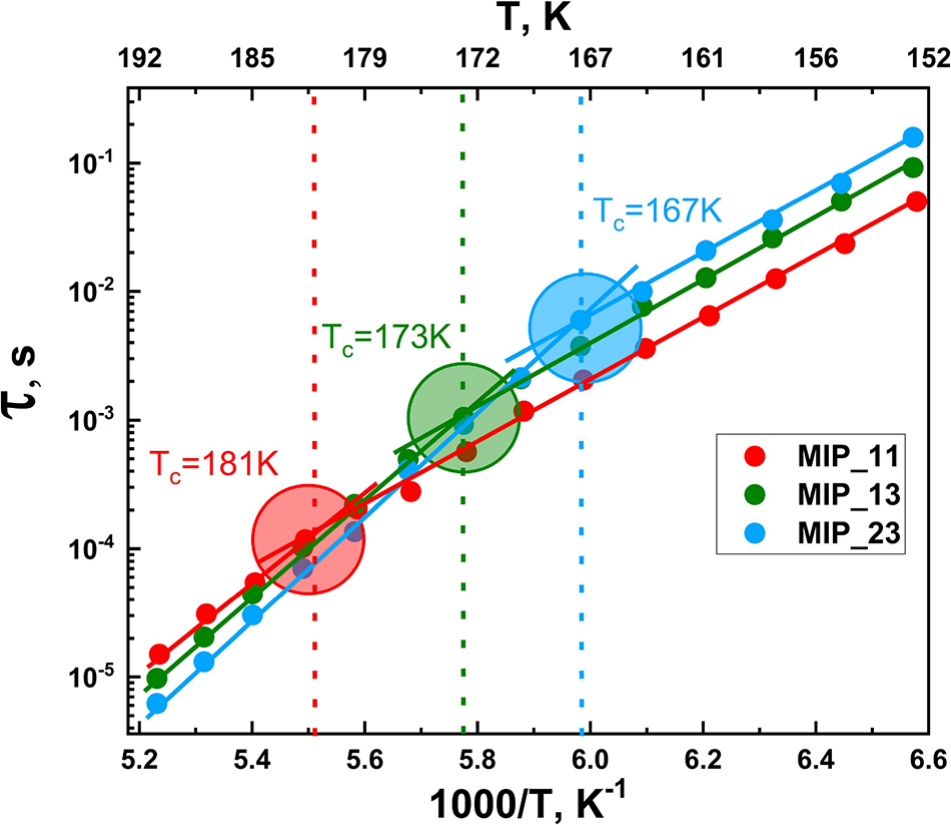
Change of the position of the relaxation time crossover of ice-like process versus inversed temperature for samples with silica gel and various hydration levels.

The calculated activation energy values for samples MIP_11, MIP_13, and MIP_23 (Figure 8) are smaller than the activation energy of bulk ice ∼60 kJ/mol.^68^ This means the amount of adsorbed water in the glasses is not enough to build ice, and water molecules organize themselves into ice-like structures. Smaller values of the activation energies reflect the deficit of water molecules required for the construction of ice clusters because water is mainly confined in the small pores of the secondary silica. At higher temperatures, MIP samples demonstrate higher activation energy values (∼70 kJ/mol). This indicates a clear change in the water dynamics after the crossover point. In the case of low humidity MAP_2, the activation energy is smaller than that of ice.^54^ Furthermore, the process related to the water adsorbed at the glass does not exhibit any crossover (MAP_2). Similar results were reported in a BDS study of weakly hydrated MCM-41 silica materials.^69^ All hydrated MIP samples containing SG inside the pores show a crossover in the dielectric relaxation map at about 180 K, similar to other confined water systems.^4^ The absence of the crossover in the SG-free samples could also be related to confinement size. Pores without SG are much bigger than confinement in SG contained samples. Whether the crossover can be associated with the matrix or the confinement size remains an open question. After providing a zoom-in of the crossover region, it is seen that the crossover position shifts with the change in the hydration level – which is shown in Figure 8.

**Figure 9:**
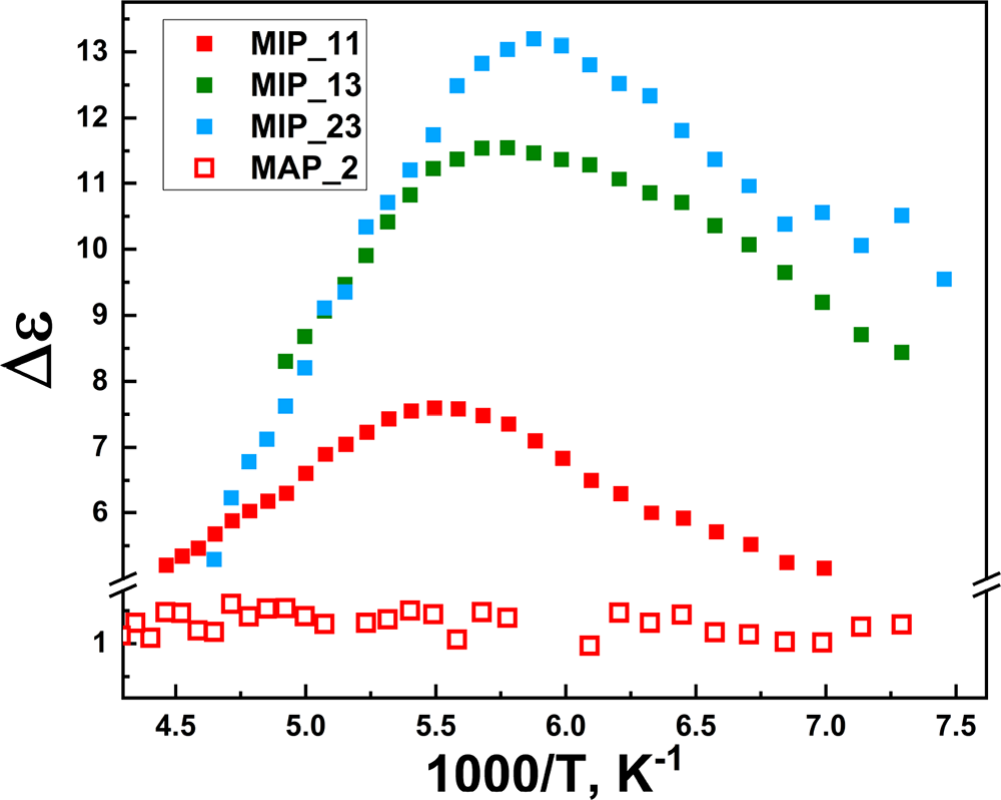
Temperature dependence of the dielectric strength for all BDS-measured hydration levels.

The temperature of the crossover point decreases when the hydration level of the sample is increased. To investigate the nature of the shift, the temperature dependence of the dielectric strength is presented in Figure 9.

For the MIP samples with various water contents, the dielectric strength shows a maximum near 180 K, whereas the MAP sample does not exhibit any maximum. This maximum shifts to lower temperatures with increasing the hydration level, resembling the findings for the crossover temperature.

An increase of Δε with temperature is usually assigned to a solid-like behavior.^70^ On the other hand, a decrease of Δε with temperature reflects a liquid-like behavior.^71^ In this scenario, we can link the change in Δε to a first-order phase transition,^71^ where the crossover observed in the *T* dependence of the relaxation times can be associated with the melting process from ice to supercooled water.

### 3.2 Differential Scanning Calorimetry

To get more evidence about the possible melting at 180 K, we measured the calorimetric response of MIP glasses hydrated at two water contents (*h* = 4.8 and *h* = 11.1 wt %). Figure S-1 in the Supporting Information shows the heat flow during the cooling scan at both hydration levels (measured at a cooling ratio of ∼30 K/min). For MIP_11, an endothermic peak (onset at ∼233 K) indicates water crystallization on cooling. In contrast, this endothermic peak is absent at *h* = 4.8 wt % (MIP_4.8, see Figure S-1 in the SI).

**Figure 10:**
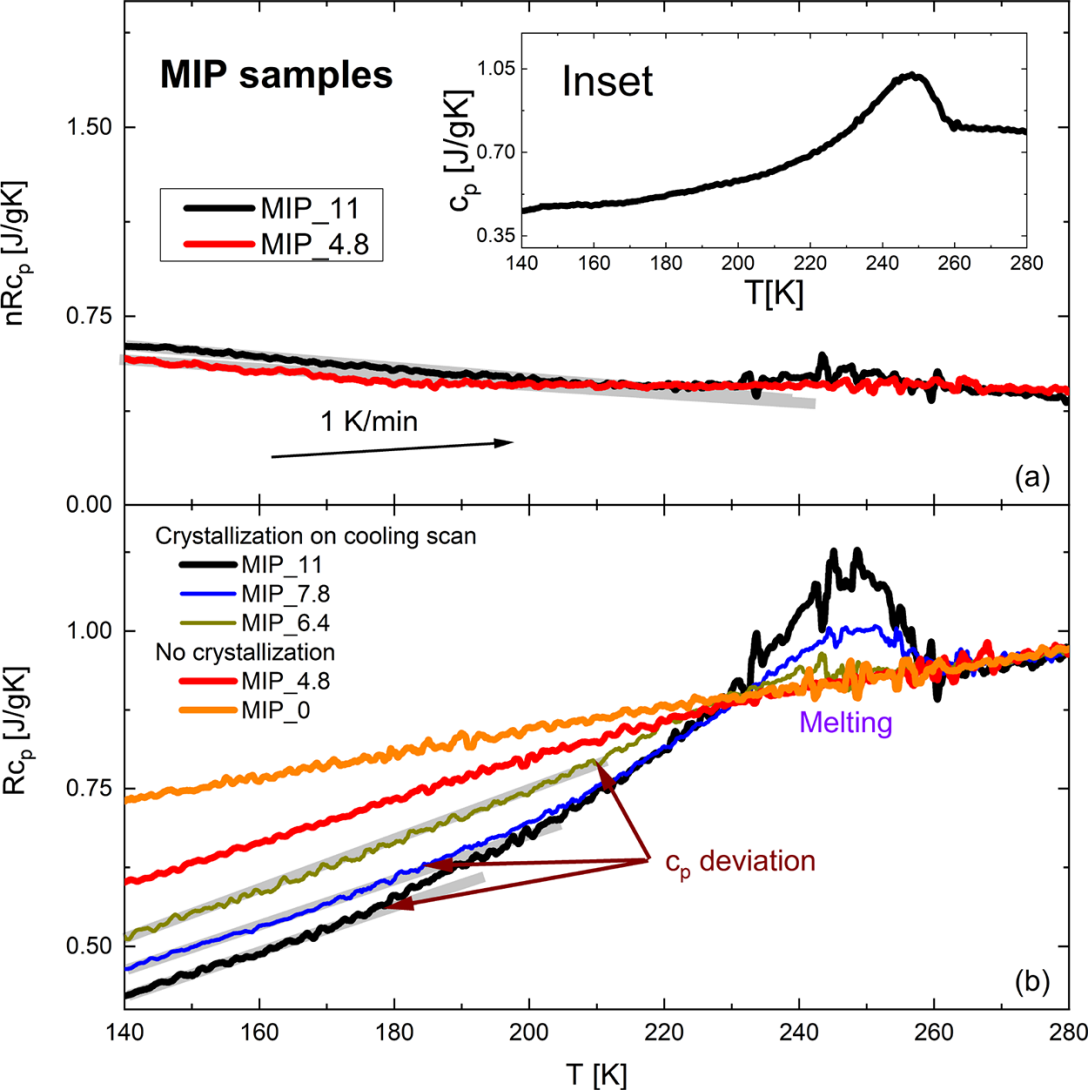
(a) Non-reversing heat capacity (nR*c*_P_) for MIP_11 and MIP_4.8 and (b) reversing heat capacity (R*c*_P_) for MIP_11, MIP_7.8, MIP_6.4, MIP_4.8, and MIP_0 (dry sample). The heating rate was 1 K/min. Inset: total heat capacity (R*c*_P_) for the same sample during the cooling scan.

Figure 10a,b shows the non-reversing (nR*c*_P_) at *h* = 4.8 and 11.1 wt % and reversing (R*c*_P_) heat capacity at *h* = 4.8, 6.4, 7.8, and 11.1 wt % during the heating scan of the samples hydrated. In addition, Figure 10b shows the baseline of the dry sample (MIP_0), which is an entirely flat signal. The inset in Figure 10a corresponds to the total heat capacity (*c*_P_). As seen in Figure 10a, the nR*c*_P_ signal for MIP_11 is flat, indicating no crystallization during the heating scan (the so-called cold-crystallization^72^) until the temperature reaches 220 K. At 220 K, a small endothermic broad peak starts related to melting.

The melting is better observed in the R*c*_P_ signal for temperatures between 230 and 260 K. In addition, a *c*_P_ deviation is produced at ∼180 K, about 50 K below the melting temperature. Note that this deviation of *c*_P_ is absent in the sample with a lower water content (*h* = 4.8 wt %) in which crystallization on cooling was not produced during the cooling scan.

Therefore, we can conclude that when water is partially crystallized on cooling, *c*_P_ shows a deviation at 180 K, the same temperature as the crossover occurs in the *T* dependence of the relaxation time.

It is important to note that the reversing *c*_P_ signal provides information related to heat capacity events, such as the glass transition and melting. It is a challenging task to distinguish between these two events when both occur in a short temperature interval (i.e., the R*c*_P_ deviation could be the consequence of a temperature extended melting).

To get more insights into the entanglement between melting and the R*c*_P_ deviation, we can calculate the percentage of water that crystallizes during cooling and compare it with the portion of water that melts during heating. For MIP_11, (12.5 ± 0.5) % of the total amount of water crystallizes on cooling. Since there are no signs of cold crystallization, we expect to calculate a melting percentage equal to the amount of water crystallized during cooling. Considering the onset of melting at 220 K, the melting percentage agrees with the amount of water crystallized during the cooling cycle. In contrast, regarding the melting starting at 180 K, the rate of water crystallization is higher ((20.3 ± 0.7) %). Therefore, we can estimate that the melting is produced at temperatures higher than 220 K and not from 180 K.

For example, considering the sample MIP_11, the percentage of amorphous water (which contributes to the overall dynamics) is 87.5%, whereas 12.5% of the total amount of water is ice and does not contribute to the overall dynamics. Thus, the *c*_P_ deviation at 180 K could be related to a glass transition of amorphous water. However, experiments in other confinements are necessary to verify this hypothesis.

Moreover, Figure 10b shows the R*c*_P_ for different water contents (7.8, 6.4, 4.8, ∼0 wt %). We can detect the deviation in R*c*_P_ for the samples with the highest water contents (11.1, 7.8, and 6.4 wt %). However, this deviation is absent for *c*_w_ = 4.8 wt % and the dried sample. As shown in Figure 10c, for 11.1 wt %, the *c*_P_ deviation occurs at around 180 K, while for 7.8 and 6.4 wt %, the temperature increases to 184 and 210 K, respectively. This behavior correlated well with the BDS results, where the crossover temperature increases with decreasing water content.

On the one hand, in the frame of the Gibbs–Thomson theory,^33^ the decrease of the Δε transition temperature with the increase of humidity represents the slight decrease of the confinement size with the increase of hydration level of the samples. Having the transition temperature from the fitting data and using the paradigm of the Gibbs–Thomson theory the confinement size is calculated by eq 1. The *r* for non-freezing layer was taken as 0.4 nm – as typical for the silica materials – and the Gibbs–Thomson coefficient *C_GT_* is equal to 56.4 K nm.^33^ The calculated confinement size is presented in Table 3.

**Table 3:** Calculated Confinement Sizes

hydration, %	*T*, K	*R*, nm	*d*, nm^a^
MIP_11	181	1.01	2.02
MIP_13	173	0.96	1.92
MIP_23	167	0.93	1.86

a *d* represents the estimated size of confinement.

The estimated size of confinement, *d*, most probably corresponds to water-accessible volume in our samples as shown in Figure 4.

### 3.3 Nuclear Magnetic Resonance

Our ^2^H NMR approaches probe the fluctuations of the quadrupolar frequencies of the D_2_O deuterons, which are approximately given by eq 3.
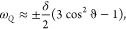
3where δ = 2π·161 kHz characterizes the strength of the associated quadrupolar interaction and ϑ describes the orientation of the O–D bonds with respect to the applied static magnetic field B_0_.

**Figure 11:**
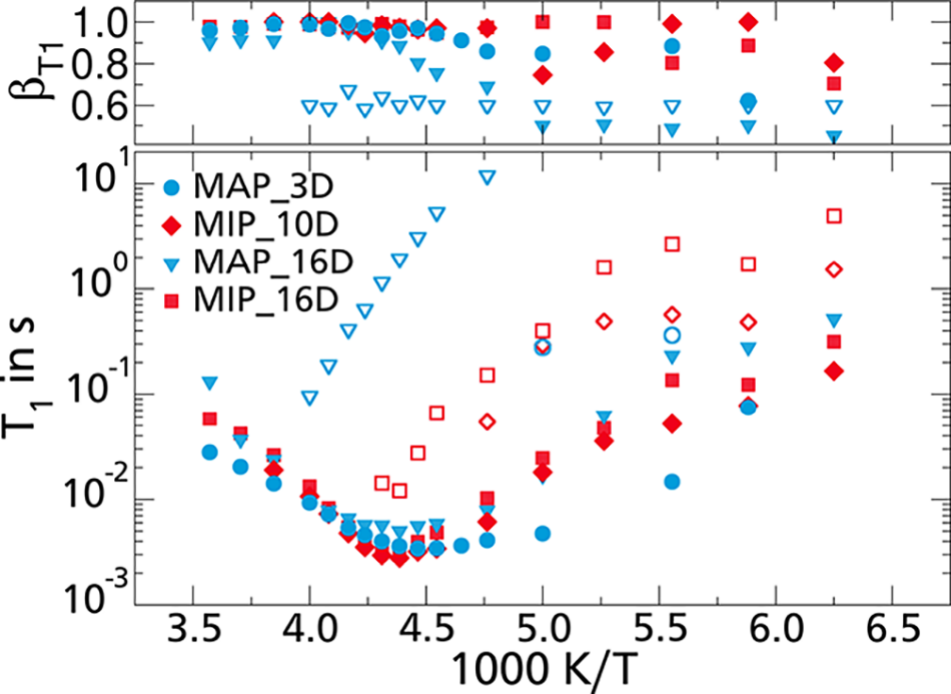
SLR time *T*_1_ and stretching parameter β_*T*1_ over temperature. The samples are specified by their hydration (D_2_O) levels. Red and blue symbols denote confinements with (MIP_10D and MIP_16D) and without (MAP_3D and MAP_16D) SG, respectively. Fast (solid symbols) and slow (open symbols) SLR components can be distinguished.

In ^2^H spin–lattice relaxation (SLR) studies, we measure the buildup of ^2^H magnetization and fit the experimental data with an adapted Kohlrausch–Williams–Watts (KWW) function, . Here, *M*_0_ denotes the equilibrium ^2^H magnetization, *T*_1_ is the SLR time, and β_T1_ is the stretching exponent. When two dynamically distinguishable water fractions coexist inside the confinements, we employ a weighted superposition of two KWW functions for the fits. The fit results are shown in Figure 11. For all studied samples, the shorter *T*_1_ time exhibits a minimum, indicating D_2_O reorientation on the nanoseconds time scale,^73^ while the longer *T*_1_ time is observed at sufficiently low temperatures and continuously increases upon cooling. Recent ^2^H SLR approaches to D_2_O in MCM-41 and SBA-15 silica pores reported similar results and showed that the faster and slower SLR components can be attributed to liquid and solid water fractions.^27,74^ Consistent with this assignment, the fast SLR component is exponential (β_*T*1_ = 1) and nonexponential (β_*T*1_ < 1) when the liquid fraction is fluid and viscous near ambient and reduced temperatures, respectively, see Figure 11, whereas the slow SLR component shows β_*T*1_ ≈ 0.6 whenever it exists. The samples with D_2_O weight fractions of ≥10% show a *T*_1_ minimum at ca. 228 K, indicating that they exhibit similar water reorientation with a correlation time of about 1 ns at this temperature. For MAP_3D, the *T*_1_ minimum associated with the liquid fraction is shifted to 222 K. Hence, the water reorientation is somewhat faster in the sample with the lower water content than in those with the higher water contents in this temperature range. A very long *T*_1_ component is observed for MAP_16D, implying that an ordered ice fraction exists in the larger MAP confinements at sufficiently high water content.

A more detailed SLR analysis is performed for the liquid water fraction in the sample MIP_10D, while this is hampered for the other samples by lower signal-to-noise ratios (3 wt %) or interfering contributions from the crystalline water fraction. For this analysis, we exploit that the ^2^H SLR time *T*_1_ is related to the spectral density *J*_2_(ω_*L*_) of water reorientation by eq 4.
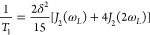
4where ω_*L*_ = 2π·46.1 MHz is the ^2^H Larmor frequency in the used magnet. To determine rotational correlation times τ from the measured *T*_1_ values, knowledge about the shape of *J*_2_(ω_*L*_) is required. Here, we assume a Havriliak–Negami (HN) function and determine its shape parameters in a two-step process. In additional ^1^H field-cycling relaxometry (FCR) studies, we first measure ^1^H *T*_1_(ω_*L*_) at low frequencies and temperatures *T* ≥ 250 K and calculate the NMR susceptibility χ_NMR_^″^(ω) ≡ ω/*T*_1_ from these data.^48^ The results in the Supporting Information (Figure S-2) reveal power laws ω_*L*_^0.84^ in the available frequency and temperature ranges, which correspond to the low-frequency flank of the loss peak associated with the HN susceptibility (eq 5).
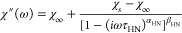
5Therefore, we fix α_HN_ = 0.84 in the ^2^H SLR analysis. This information can then be exploited to assess the second shape parameter of the HN function from the minimum *T*_1_ value,^73^ yielding β_HN_ = 0.44. Using the thus determined HN spectral density in eq 4, we obtain the HN time constants τ_HN_ from the measured *T*_1_ values. From these time constants and the shape parameters, we calculate the HN peak correlation times according to eq 6.^75^
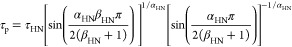
6

**Figure 12:**
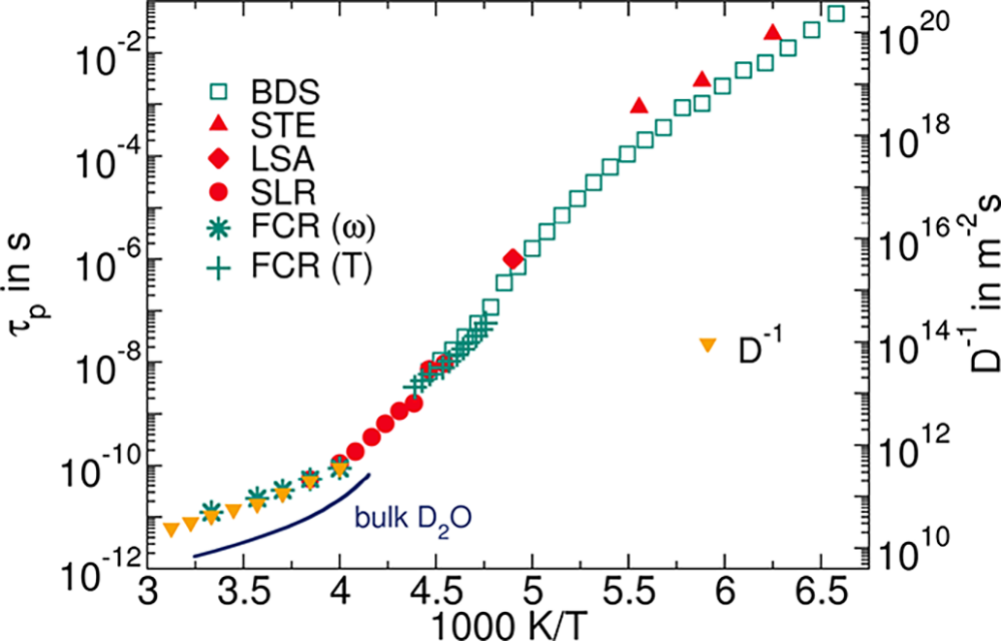
Temperature-dependent correlation times τ_P_ for MIP_10 (green and yellow symbols) and MIP_10D (red symbols) obtained from ^2^H SLR, LSA and STE, and ^1^H FCR. In the latter case, results from *T*_1_(ω) and *T*_1_(T) analyses are shown, see the SI (Figures S-2 and S-3). For comparison, the BDS data from Figure 7 and the inverse self-diffusion coefficients *D*^–1^ from ^1^H SFG diffusometry are included. Moreover, rotational correlation times of bulk D_2_O are displayed as a line.^76^

Figure 12 shows the correlation times τ_p_ of MIP_10D obtained from this ^2^H SLR analysis. The SLR data reveal a non-Arrhenius temperature dependence above 220 K and agree with the BDS results near 220 K. To scrutinize the ^2^H SLR analysis, we also determine correlation times from the ^1^H FCR data for MIP_10, explicitly, from the shift factors used to collapse the power laws ω_*L*_^0.84^ of *T*_1_(ω) at various temperatures onto a master curve [FCR (ω)] and from a determination of *T*_1_(T) minima for various fixed frequencies ω [FCR (T)], see the Supporting Information (Figures S-2 and S-3).

In Figure 12, we see that the ^1^H and ^2^H correlation times agree in the common temperature range and that the former extend the latter to higher and lower temperatures. Moreover, it can be seen that water reorientation is about a factor of 5 slower in MIP_10D than in the bulk. In addition, we display inverse self-diffusion coefficients *D*^–1^ obtained for MIP_10 from ^1^H SFG diffusometry in Figure 12. We observe that τ_p_ and *D*^–1^ follow the same non-Arrhenius temperature dependence in the common temperature range. Altogether, the agreement of the ^1^H, ^2^H, and BDS data (i) confirms the validity of our SLR analyses, (ii) indicates that there are no marked differences between H_2_O and D_2_O reorientation at sufficiently high temperatures, and (iii) shows that the SLR and BDS correlation times characterize the structural α-relaxation of the confined water in the temperature range above 220 K. In particular, the consistency of correlation times, diffusion coefficients, and bulk data near ambient temperatures implies that the ice-like (first, ν/main) process involves viscosity-related dynamics above the crossover temperature *T*_c_.

**Figure 13:**
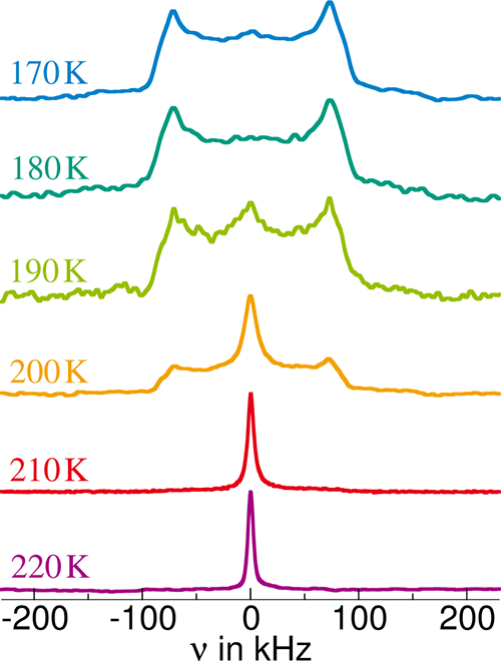
^2^H NMR spectra of MIP_10D at the indicated temperatures.

^2^H NMR line-shape analysis (LSA) provides access to D_2_O reorientation on the microseconds time scale. Figure 13 presents the temperature-dependent ^2^H NMR spectra of MIP_10D. We see that the narrow liquid-like (Lorentzian) line at *T* ≥ 220 K evolves into a broad solid-state (Pake) spectrum when decreasing the temperature to *T* ≤ 180 K. This indicates that the time scale of the D_2_O reorientation crosses the microsecond time window of the LSA approach. Moreover, the Lorentzian shape implies that the rotational motion is essentially isotropic at least above the line-shape transition, further confirming α-like water dynamics in this temperature range. Near 200 K, the ^2^H NMR spectrum can be described as a weighted superposition of Lorentzian and Pake contributions, indicating that fast (τ ≪ 1 μs) and slow (τ ≫ 1 μs) water molecules coexist at a given temperature. This finding is consistent with the above broad BDS and SLR susceptibilities, which implies the existence of broad distributions of correlation times for the confined water. Such line-shape transitions are accompanied by a minimum of the signal intensity in the applied solid-echo sequence.^29^ Explicitly, the solid-echo signal minimum occurs at 204 K, indicating τ = 1 μs in very good agreement with the above BDS results, see Figure 12.

**Figure 14:**
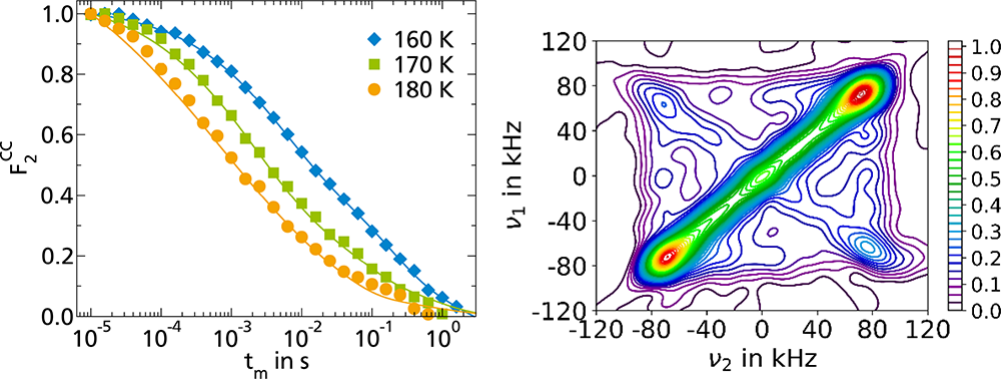
Results from 2D ^2^H NMR studies on MIP_10D: (left) Correlation function *F*_2_^*cc*^(*t_m_*) from STE experiments at the indicated temperatures. The lines are fits with eq 8, revealing a faster decay due to water reorientation and a slower decay due to spin relaxation. (right) 2D ^2^H NMR spectrum for a mixing time of *t_m_* = 30 ms at 170 K.

Finally, we perform two dimensional (2D) ^2^H NMR experiments in both the time domain and the frequency domain to investigate slow D_2_O reorientation at low temperatures. ^2^H stimulated-echo (STE) experiments allow us to measure rotational correlation functions (7),^27,73,77^
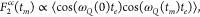
7which correlate the quadrupolar frequencies ω_*Q*_ during short evolution times *t_e_* = 5 μs, which are separated by a variable mixing time *t_m_*. Results for MIP_10D are shown in Figure 14 (left panel). *F*_2_^*cc*^(*t_m_*) shows nonexponential decays, which shift to longer times when the temperature is decreased, reflecting the slowdown of D_2_O reorientation. For a quantitative analysis, we fit the STE decays to eq 8.
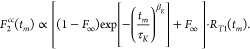
8Hence, we interpolate the correlation loss due to water reorientation with a KWW function described by the time constant τ_*K*_ and the stretching parameter β_*K*_, we introduce *F*_∞_ to account for a possible residual correlation due to either anisotropic water reorientation or an immobile water fraction, and we consider an additional long-time damping due to SLR during *t_m_*, *R*_*T*1_(*t_m_*), which is known from independent SLR measurements and fixed in the STE analysis. To enable straightforward comparison with the BDS results, we calculate peak correlation times τ_p_ from the resulting fit parameters τ_*K*_ and β_*K*_ (9),^78^
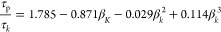
9

Revisiting Figure 12, we find that the STE results are also in reasonable agreement with the BDS findings. Explicitly, the STE correlation times are about a factor of three longer than the BDS counterparts but both data sets exhibit the same temperature dependence, which can be described by an Arrhenius law with an activation energy of 47 kJ/mol. Thus, NMR and BDS probe the same reorientation process of water also in the Arrhenius regime at *T* < 180 K, consistent with previous ^2^H STE results for D_2_O in hard MCM-41 pores.^27,77^ For the residual correlation, the fits yield *F*_∞_ ≈ 0.2, again consistent with previous findings for confined water at low temperature.^74^ Thus, the water reorientation destroys large parts of the orientation correlation and, hence, it can be characterized as a large-amplitude motion. However, based on the available STE data, we cannot decide whether the finite residual correlation of *F*_∞_ ≈ 0.2 results from some anisotropy of the water reorientation, as was discussed for water in protein matrices at low temperatures,^29,79,80^ or from some slow water fraction, which does not show reorientational motion in the experimental time window.

More detailed insights into the geometry of the reorientation process are available from 2D ^2^H NMR spectra, which correlate the NMR frequencies of the deuterons before and after the mixing time *t_m_*,^81^ explicitly, 2πν_1_ = ω_*Q*_(0) and 2πν_2_ = ω_*Q*_(*t_m_*). Figure 14 (right panel) shows the 2D ^2^H NMR spectrum S_2_(ν_1_,ν_2_; *t*_m_) of MIP_10D at 170 K. It features diagonal intensity (ν_1_ = ν_2_) and off-diagonal intensity (ν_1_ ≠ ν_2_), resulting from water molecules, which are static (τ > *t_m_*) and mobile (τ < *t_m_*) during the mixing time, respectively. The off-diagonal intensity is box-shaped, indicating a quasi-isotropic reorientation process. By contrast, the off-diagonal intensity would be pooled in elliptical features if there were rotational jumps about defined angles^82^ or it would be limited to regions near the diagonal if the reorientation process was restricted.^83^ In the Supporting Information (Figure S-4), we show that the box-like shape of the off-diagonal intensity remains unchanged in the temperature range 160–180 K, while the off-diagonal intensity decreases with respect to the diagonal intensity upon cooling, reflecting the slowdown of the water reorientation. Hence, the 2D ^2^H NMR spectra indicate that the water reorientation is quasi-isotropic even in the Arrhenius regime below 180 K.

## 4 Conclusions

Confined water relaxation was studied by broadband dielectric spectroscopy (BDS), nuclear magnetic resonance (NMR), and differential scanning calorimetry (DSC). Water was confined in porous glasses of two types: MIP with silica gel (SG) and MAP without SG. The combination of structures provides nanosized geometrical confinements and allows studying water confined by rigid/hard (glass) and soft (SG) matrices. MAP-type samples have pores of about 10–35 nm, and MIP-type samples have confinements sizes of around 2.5–3 nm. When the former pores are loaded with small weight fractions of water, all water molecules reside in a thin layer at the inner glass surfaces, whereas a freezable water fraction exists at higher loadings.

The MIP samples showed the relaxation response of two water species. One is bound to the glass walls, and the other one to the SG hydration centers. Two dynamically different water fractions were also found in different regions of silica mesopores.^6^

BDS and NMR techniques clearly showed that water exhibits a crossover point at around 180 K when confined to about 2 nm with secondary silica (MIP samples). In addition, DSC showed a *c*_P_ deviation at the same temperature. Furthermore, it was found that the crossover temperature depends on the hydration levels: the higher the hydration level, the lower the crossover temperature.

The interpretation of the crossover temperature can include two points of view. On the one side, within the framework of the Gibbs–Thomson theory, it can be concluded that the crossover is related to the melting of ice crystals because the melting point is highly dependent on the pore diameter as observed by NMR, BDS and DSC.

Conversely, if the *c*_P_ deviation is interpreted as a water glass transition of confined water, the crossover temperature could be interpreted as the change between a solid-like to a liquid-like behavior. Consistently, the NMR results indicate that the probed water reorientation is essentially isotropic and, at least above the crossover temperature, related to long-range diffusion, leading to the typical non-Arrhenius temperature dependence of the viscosity-related α-relaxation. In addition, BDS parameters also vary as expected from a solid-like behavior to a liquid- like behavior as the temperature increases.

The sample with a larger confinement size and low water content does not demonstrate the specific crossover feature. The present data do not allow us to decide whether this difference results from the fact that water resides in soft (MIP) and hard (MAP) confinements, respectively, or from the fact that there are different water contents and, hence, that different fractions of water molecules form hydrogen bonds with the silica matrix. Previous studies reported that the crossover occurs in both hard and soft confinements provided sufficient water so that water–water interactions are relevant. Nevertheless, further investigation is required to decide between these two situations. In particular, it is necessary to find exactly where the confined water is located in the MIP samples—inside the SG, between the SG particles, or at the inner glass surface. The tiny ice crystal size does not allow X-ray diffraction analysis (XRD), and neutron scattering techniques could be helpful in this case. Finally, more hydration levels and temperatures should be studied to prove the crossover temperature dependence on hydration level.

## Supporting Information Available

Calorimetric measurements, ^1^H field cycling relaxometry, and 2D ^2^H NMR spectra (PDF)

## Abbreviations

MIP: microporous
MAP: macroporous
SG: silica gel
BDS: broadband dielectric spectroscopy
NMR: nuclear magnetic resonance
DSC: differential scanning calorimetry
PVP: polyvinylmethylether
PGME: propylene glycol monomethylether
SLR: spin–lattice Relaxation
KWW: Kohlrausch–Williams–Watts
CC: Cole–Cole function
STE: stimulated-echo experiment
FCR: field-cycling relaxometry
SFG: static field gradient
SEM: scanning electron microscope
TMDSC: temperature-modulated differential scanning calorimetry
VFT: Vogel–Fulcher–Tammann
