# Supplementary material for: The Nature of the Low-Temperature Crossover of Water in Hard Confinement
Source: J Phys Chem B. 2023 May 25;127(22):5128–40. doi: 10.1021/acs.jpcb.3c00747 (PMC10258804; doi:10.1021/acs.jpcb.3c00747)
Supplement: Supplementary file 1 — jp3c00747_si_001.pdf [file jp3c00747_si_001.pdf]

The nature of the low temperature crossover of water in hard confinement

*Yael Beilinson[1], Verena Schiller[2], Julia Regentin[2], Jorge H. Melillo[3], Anna Greenbaum[1,4], Tatiana Antropova[5], Silvina Cerveny[3,6] *, Michael Vogel[2], Yuri Feldman[1]*

[1]The Hebrew University of Jerusalem, Department of Applied Physics, Edmond J. Safra Campus, Jerusalem 9190401, Israel

[2]Institut für Physik kondensierter Materie, Technische Universität Darmstadt, Hochschulstraße 6, 64289 Darmstadt, Germany

[3]Donostia International Physics Center (DIPC), Paseo Manuel de Lardizabal 4, 20018 San Sebastian, Spain

[4]The Hebrew University of Jerusalem, Racah Institute of Physics, Edmond J. Safra Campus, Jerusalem 9190401, Israel

[5]Grebenshchikov Institute of Silicate Chemistry, Russian Academy of Sciences, Makarova emb., 2, Saint-Petersburg 199034, Russia

[6]Centro de Física de Materiales (CFM CSIC/EHU) - Material Physics Centre (MPC), Paseo Manuel de Lardizabal 5, 20018 San Sebastian, Spain

1. Calorimetric Measurements

Figure S-1 represents heat flow as a function of the temperature of MIP samples at two hydration levels.

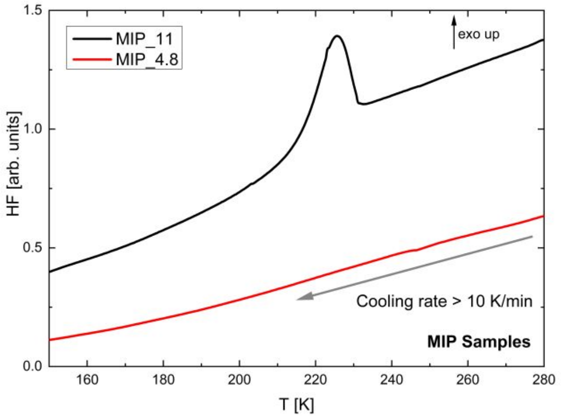

Figure S-1. Heat flow as a function of the temperature of MIP samples at two hydration levels.

2. [1]H field cycling relaxometry

In ^{1}H field-cycling relaxometry (FCR), the ^{1}H spin-lattice relaxation (SLR) times T_1 are measured in a broad range of Larmor frequencies ω utilizing a rapidly switchable electromagnet [33, 75]. Exploiting the time-honored relations between spin relaxation and molecular dynamics, the measured $T_1(\omega)$ dispersions provide straightforward access to the spectral densities $J_2(\omega)$, which describe the fluctuations of the dipolar interactions between the protons and, thus, the underlying molecular motion. Explicitly, for ^{1}H, the Bloembergen-Purcell-Pound (BPP) relation reads (10) [76].

$$\frac{1}{T_1} = C[J_2(\omega) + 4J_2(2\omega)] \,, \quad (10)$$

where the coupling constant C describes the strength of the dipolar interactions.

Due to the strong distance dependence, the dipolar interactions are governed by the contributions from the proton pairs of the water molecules in our case and, hence, the fluctuations largely reflect the reorientation of the proton-proton internuclear vector of the water molecules. Although the spectral densities $J_2(\omega)$ contain the relevant information about molecular reorientation, it proved to be advantageous to switch to a susceptibility representation for detailed analyses [34, 77]. Specifically, an NMR susceptibility $\chi''_{NMR}(\omega)$ can be defined according to (11).

$$\frac{\omega}{T_1(\omega)} = C\left[\omega J_2(\omega) + 4\omega J_2(2\omega)\right] =$$

$$= C\left[\chi''(\omega) + 2\chi''(2\omega)\right] \equiv \chi''_{NMR}(\omega) \quad (11)$$

Figures S-2 and **S-3** display results of ^{1}H FCR measurements on the microporous glass with silica gel and a water content of 10 wt% (MIP_10). In **Figure S-2**(**a**), we show $\chi''_{NMR}(\omega)$ at various temperatures. The available frequency and temperature ranges are restricted by the facts that T_1 becomes comparable to the field-switching times of the used electromagnet at lower temperatures, interfering with reliable measurements, and processes other than water reorientation start to dominate the $T_1(\omega)$ dispersions at higher temperatures [78, 79], as can be seen from the flattening

out of $\chi''_{NMR}(\omega)$ at low frequencies. As a result of these restrictions, a susceptibility maximum is not observed in the available frequency and temperature ranges but the ^{1}H FCR measurements merely provide access to the low-frequency flank of the expected peak. Nevertheless, we see that $\chi''_{NMR}(\omega)$ shifts to lower frequencies upon cooling and that the low-frequency flanks appear to have a similar slope at sufficiently high frequencies. In **Figure S-2**(**b**), the latter aspect is investigated in more detail by horizontally shifting the NMR susceptibilities at various temperatures for best possible overlap. It can be seen that the data collapse onto a master curve, which implies that the low-frequency flank of the susceptibility peak is described by a power law $\omega^{0.84}$ independent of temperature. Hence, the Cole-Davidson function does not correctly characterize the NMR susceptibilities of the confined water.

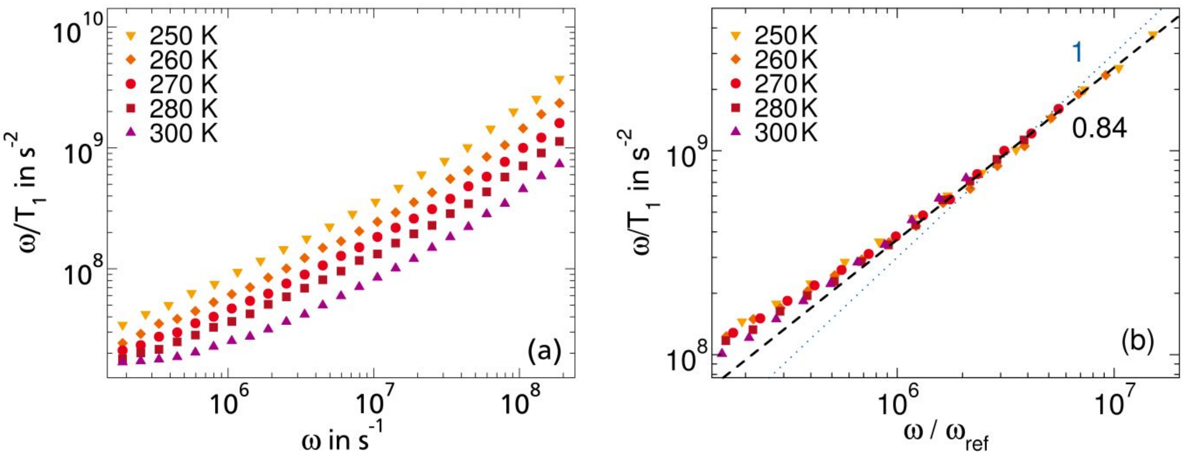

Figure S-2. (**a**) NMR susceptibility $\chi''_{NMR}(\omega)$ from ^{1}H FCR studies on the microporous glass with silica gel and water content of 10 wt% (MIP_10) at the indicated temperatures. (**b**) NMR susceptibility master curve was obtained by shifting the data at various temperatures along the frequency axis. The dashed and dotted lines indicate slopes of 0.84 and 1.0 in the chosen double logarithmic representation.

Rather, we utilize a Havriliak-Negami function with a low-frequency shape parameter of α_{HN} = 0.84 in the main text. The shift factors used to construct the master curve yield correlation times provided the value at a suitable reference temperature is known. Here, we employ the reference value $\tau = 54$ ps at 260 K, as obtained from the ^{2}H SLR analysis in the main text. The obtained correlation times are included in **Figure 12** of the main text and labeled as 'FCR (ω)'.

Alternatively, ^{1}H FCR can be used to measure $T_1(T)$ for various fixed Larmor frequencies ω. **Figure S-3** shows exemplary data for the microporous glass with silica gel and water content of 10 wt%. We see $T_1(T)$ minima, which shift to lower temperatures when the frequency is reduced. Correlation times are available from these results when we determine the $T_1(T)$ minima from parabolic fits and exploit that $\omega\tau = 0.616$ is valid at the respective temperatures [76]. The resulting correlation times are also included in **Figure 12** of the main text and labeled as 'FCR (T)'.

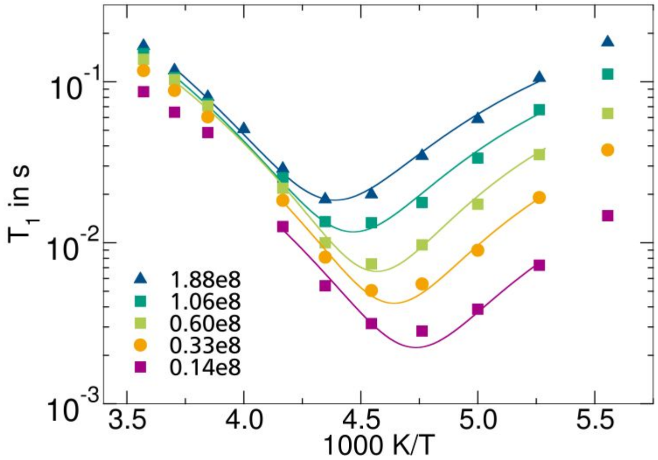

Figure S-3. [1]H SLR times $T_1(T)$ at the indicated fixed frequencies ω from ^{1}H FCR studies on the microporous glass with silica gel and a water content of 10 wt% (MIP_10). The lines are parabolic fits.

3. 2D ^{2}H NMR spectra

2D NMR spectra correlate the NMR frequencies of a nuclear spin before (ν_1) and after (ν_2) a mixing time t_m. Specifically, the 2D NMR spectral intensity $S_2(\nu_1,\nu_2;\ t_m)$ is proportional to the joint probability density $P_2(\nu_1,\nu_2;\ t_m)$ of finding the frequencies ν_1 and ν_2 during the respective time periods [71]. Our ^{2}H NMR approach probes the quadrupolar frequencies; thus, $2\pi\nu_1$ and $2\pi\nu_2$ correspond to $\omega_Q(t=0)$ and $\omega_Q(t=t_m)$, respectively, which, in turn, reflecting the molecular orientations at these times. Therefore, the spectral intensity $S_2(\nu_1,\nu_2;\ t_m)$ will be restricted to the diagonal $\nu_1 = \nu_2$ if $\tau \gg t_m$ and, hence, molecular reorientation during the mixing time can be

neglected. By contrast, off-diagonal intensity $\nu_1 \neq \nu_2$ indicates changes of the molecular orientations during t_m. Thereby, the shape of the off-diagonal intensity is determined by the geometry of the reorientation process [71]. Explicitly, isotropic rotational motion distributes the spectral intensity in the ν_1- ν_2 plane, leading to a box-shaped pattern. By contrast, rotational jumps about defined angles result in elliptical features and restricted rotational motions limit the spectral intensity to the region near the diagonal.

Figure S-4 shows the 2D ^{2}H NMR spectra of the microporous glass with silica gel and a water content of 10 wt% for a mixing time of $t_m = 30$ ms and various temperatures. $S_2(\nu_1, \nu_2; t_m)$ shows a lower relative amount of off-diagonal intensity at lower temperatures reflecting the fact that less water molecules are mobile during the mixing time when the temperature is reduced. However, the shape of the off-diagonal intensity remains unaltered when cooling from 180 K to 160 K Therefore, the behavior observed in $\Delta\varepsilon$ is consistent with the formation of disordered ice at temperatures lower than 180 K and supercooled water at higher temperatures. In this scenario, the crossover observed in the T-dependence of the relaxation times can be associated with the melting process from ice to supercooled water.

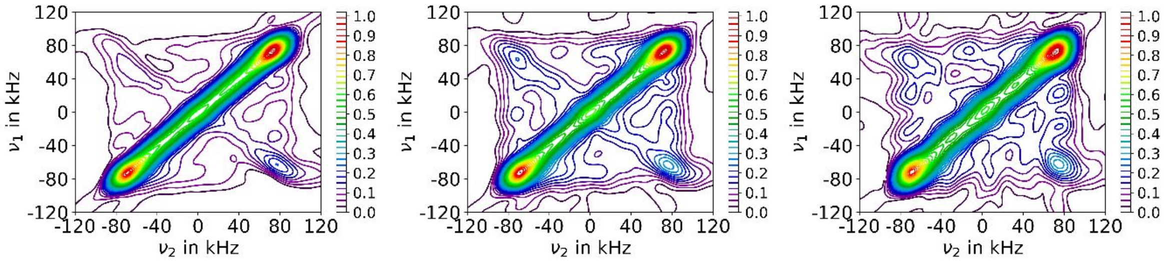

Figure S-4. 2D ^{2}H NMR spectra of the microporous glass with silica gel and a water content of 10 wt% (MIP_10) at (left) 160 K, (middle) 170 K, and (right) 180 K. The mixing time was set to t_m = 30 ms at all temperatures.

At all studied temperatures, $S_2(\nu_1, \nu_2;\ t_m)$ shows a box-like shape, indicating that water reorientation is quasi-isotropic even below the crossover observed for the temperature-dependent correlation times in the main text. The 2D ^{2}H NMR spectrum at 170 K is also shown in the main text.
